# Mechanisms of Regulation of the Chemokine-Receptor Network

**DOI:** 10.3390/ijms18020342

**Published:** 2017-02-07

**Authors:** Martin J. Stone, Jenni A. Hayward, Cheng Huang, Zil E. Huma, Julie Sanchez

**Affiliations:** 1 Infection and Immunity Program, Monash Biomedicine Discovery Institute, Monash University, Clayton, VIC 3800, Australia; jenni.hayward@monash.edu (J.A.H.); cheng.huang@monash.edu (C.H.); zil.huma@monash.edu (Z.E.H.); julie.sanchez@monash.edu (J.S.); 2 Department of Biochemistry and Molecular Biology, Monash University, Clayton, VIC 3800, Australia

**Keywords:** chemokine, chemokine receptor, regulation, binding, expression, glycosaminoglycan, post-translational modification, oligomerization, signaling, inhibitor

## Abstract

The interactions of chemokines with their G protein-coupled receptors promote the migration of leukocytes during normal immune function and as a key aspect of the inflammatory response to tissue injury or infection. This review summarizes the major cellular and biochemical mechanisms by which the interactions of chemokines with chemokine receptors are regulated, including: selective and competitive binding interactions; genetic polymorphisms; mRNA splice variation; variation of expression, degradation and localization; down-regulation by atypical (decoy) receptors; interactions with cell-surface glycosaminoglycans; post-translational modifications; oligomerization; alternative signaling responses; and binding to natural or pharmacological inhibitors.

## 1. Introduction

It has long been recognized that a hallmark feature of the inflammatory response is the accumulation of leukocytes (white blood cells) in injured or infected tissues, where they remove pathogens and necrotic tissue by phagocytosis and proteolytic degradation. A major advance in our understanding of the molecular mechanisms underlying leukocyte migration (trafficking) was the discovery of the chemokines and chemokine receptors [1,2,3]. Chemokines are small proteins expressed in tissues during normal immune surveillance or in response to injury or infection. They subsequently bind and activate chemokine receptors, G protein-coupled receptors (GPCRs) imbedded in the cell membranes of leukocytes, thereby inducing leukocyte adhesion to the vessel wall, morphological changes, extravasation into the inflamed tissue, and chemotaxis along the chemokine gradient to the site of injury or infection [1].

In addition to their roles in leukocyte trafficking, chemokine activation of chemokine receptors can give rise to a variety of additional cellular and tissue responses, including proliferation, activation, differentiation, extracellular matrix remodeling, angiogenesis, and tumor metastasis [4,5,6,7]. Moreover, two major pathogens (HIV-1 and the malarial parasite *Plasmodium vivax*) have evolved mechanisms to utilize chemokine receptors to invade host cells [8,9], and other viruses or parasites produce proteins that inhibit chemokines or their receptors so as to suppress the host immune response. Due to their central roles in inflammation, many chemokine receptors (and to a lesser extent chemokines) have been identified as potential therapeutic targets in a wide range of inflammatory diseases [10].

**Figure 1:**
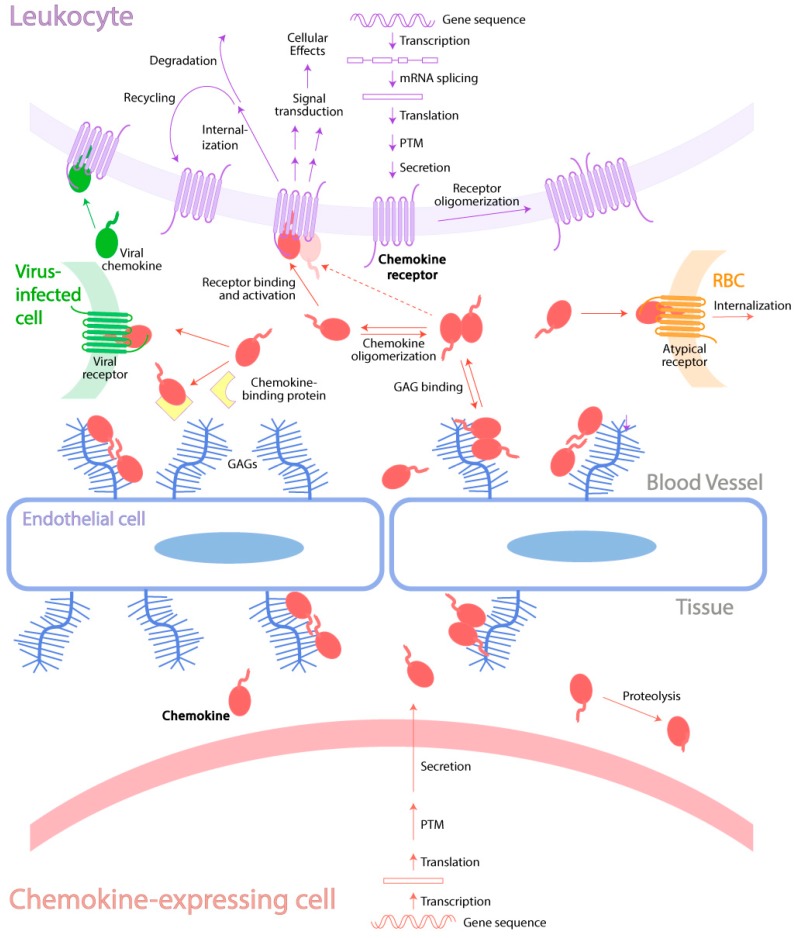
Schematic overview of regulation mechanisms of the chemokine-receptor network. Abbreviations: PTM: post-translational modification; RBC: red blood cell. Arrows in red, purple, green and orange indicate processes involving chemokines, chemokine receptors, viral chemokines and atypical receptors, respectively.

Considering the importance of chemokine-receptor interactions in responding to environmental threats but the potential risks of excessive leukocyte recruitment, it is perhaps not surprising that numerous mechanisms (summarized in Figure 1) have evolved to regulate the activities of both chemokines and their receptors. These mechanisms may involve modulation of the concentrations of these proteins in specific tissues, changes in their molecular structures, or alteration of their interactions, all of which will influence leukocyte trafficking. This Special Issue of the *International Journal of Molecular Sciences* focuses on the natural and pharmacological mechanisms by which the activities of chemokines and their receptors can be regulated. In this review article, we provide an overview and highlight illustrative examples of these biochemical and cellular mechanisms.

## 2. The Chemokine and Chemokine Receptor Protein Families

### 2.1. The Chemokine Protein Family

**Figure 2:**
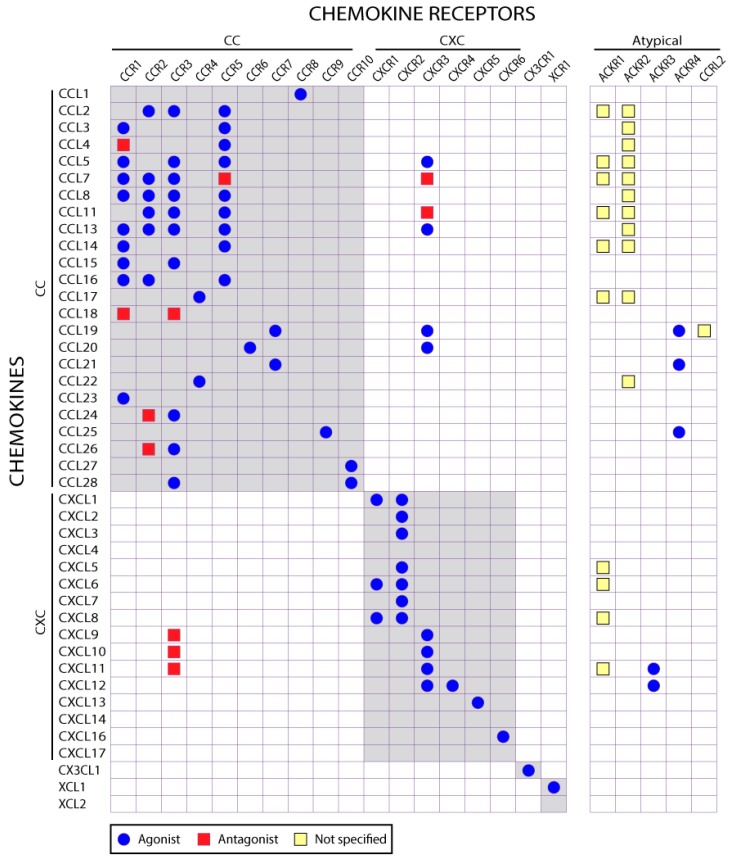
The human chemokine-receptor network. Human chemokines and receptors are listed with symbols indicating whether they are specified as agonists or antagonists (or not specified) in the IUPHAR database. Note that, although CXCL1 is listed as a CXCR1 agonist in IUPHAR, the database reference suggests that it is actually an antagonist [11].

Chemokines are small proteins (usually ~70–80 amino acid residues) with conserved sequence and structural features. The human genome and other mammalian genomes each encodes approximately 50 different chemokines (Figure 2), which are classified into two major subfamilies (CC and CXC) and two minor subfamilies (CX3C and XC) based on the spacing of conserved cysteine residues approximately 10 residues from the N-terminal end of the peptide chain. In the CC, CXC, and CX3C subfamilies, the two Cys residues (which form disulfide bonds to other conserved Cys residues within the chemokine) are separated by 0, 1, and 3 residues, respectively, whereas in the XC subfamily the second Cys (and its disulfide bond partner) are absent from the sequence. Chemokines are designated according to their subfamily classification by systematic names composed of a prefix (CCL, CXCL, CX3CL, or XCL; “L” signifies a ligand as opposed to a receptor) followed by an identifying number. However, most chemokines also have common or historical names relating to their earliest characterized functions. Herein we use the systematic names but also give the common name (or abbreviation) of each chemokine when it is first mentioned.

In addition to the sequence classification, chemokines have also been categorized based on their biological roles. Whereas most chemokines are considered proinflammatory because their expression is induced in response to tissue damage, a small subset are classified as constitutive as they are expressed in healthy tissue and play roles in maintaining normal immune functions such as lymphocyte homing to the bone marrow.

### 2.2. The Chemokine Receptor Protein Family

Chemokine receptors are GPCRs—integral membrane proteins composed of seven transmembrane helical segments. Different subsets of leukocytes express different arrays of chemokine receptors enabling them to respond to the appropriate ligands. Upon binding to their cognate chemokine ligands, the receptors undergo conformational changes giving rise to activation of intracellular effectors (G proteins or β-arrestins), initiation of signal transduction pathways and, ultimately, cellular responses. As discussed below, some chemokines may bind to receptors without inducing transmembrane signals and a few receptors (known as atypical receptors) are not G protein-coupled but still bind to chemokines.

Mammalian genomes each encode approximately 20 chemokine receptors (Figure 2). Because the receptors were discovered after the chemokines and most of them are selective for members of one chemokine subfamily, they are classified according to the subfamily of chemokines to which most of their ligands belong. Thus, receptors are named using the prefixes CCR, CXCR, CX3CR, and XCR followed by an identifying number.

### 2.3. Selectivity of Chemokine-Receptor Interactions

Most chemokines bind and activate several receptors. Similarly, most chemokine receptors respond to multiple chemokine ligands. This selectivity of recognition is an intrinsic property of the chemokine-receptor pair, i.e., a consequence of their amino acid sequences. However, selectivity can be altered by modification of the proteins (see below). Initially, the existence of multiple ligands for the same receptor was thought to represent biochemical redundancy. However, it is now often argued to be a sophisticated strategy enabling fine tuning of leukocyte responses to different inflammatory stimuli.

Figure 2 illustrates the complexity of chemokine-receptor recognition and selectivity. Notably, others reviews of chemokines and receptors often show similar illustrations, but typically these diagrams all differ from each other in their details, depending on the source of the information. Indeed there are numerous apparent inconsistencies in the literature on chemokine-receptor recognition, indicating that conclusions regarding agonist or antagonist activity are often dependent on such variables as cell type, growth conditions, source of chemokine, and assays used.

An important consequence of multiple ligands activating the same receptor is that, if they are present in the same tissues, they would be expected to compete with each other. Thus, the degree of saturation of a particular receptor by a particular cognate chemokine will depend not only on the available concentrations of receptor and chemokine but also on the available concentrations of other chemokines to which the receptor binds and other receptors to which the chemokine binds. Moreover, in addition to being dependent on the degree of receptor saturation (equilibrium binding), transmembrane signaling may also be influenced by the association and dissociation rates (kinetics) of chemokine-receptor pairs. At present, little is known about such kinetic effects. In summary, even without considering the many additional mechanisms of regulation discussed below, the complexity of the chemokine-receptor network makes it very difficult to draw direct inferences about receptor activation simply from measurements of chemokine concentrations and receptor expression levels.

### 2.4. Structural Basis of Chemokine-Receptor Recognition

The three-dimensional (3D) structures of many chemokines have been determined by NMR spectroscopy and/or X-ray crystallography [12] and the structures of several chemokine receptors have now been solved, including two with bound chemokines [13,14]. Numerous mutational studies have identified functionally important elements of both chemokines and receptors.

**Figure 3:**
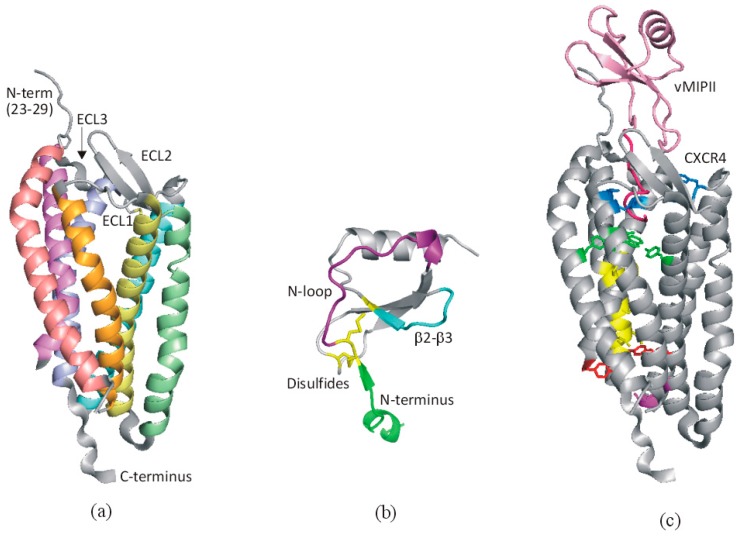
Structural basis of chemokine-receptor recognition. (**a**) One monomer unit of the receptor CXCR4 (PDB code 4RWS [14]) with extracellular regions labeled; transmembrane helices are colored salmon (I), orange (II), yellow (III), green (IV), turquoise (V), violet (VI), and magenta (VII). (**b**) A typical chemokine monomeric unit (CCL2/MCP-1, PDB code 1DOK [18]) highlighting the critical regions for receptor recognition. (**c**) Structure of CXCR4 bound to vMIPII (PDB code 4RWS [14]) showing the chemokine in pink (N-terminal region in hot pink) and the receptor in gray, with residues proposed to be involved in transmembrane signaling [19] colored according to their putative roles: blue, chemokine engagement; green, signal initiation; yellow, signal propagation; red, microswitch residues; magenta, G protein coupling. In panels (**a**,**c**) residues 1-22 are not shown as they were not modeled in the crystal structure.

Like other GPCRs, chemokine receptors consist of seven transmembrane helices aligned approximately parallel to each other and packed together in a compact bundle (Figure 3a) [15,16,17]. The extracellular face of the receptor includes an extended, largely unstructured N-terminal region and three connecting loops (extracellular loops, ECL1, 2, and 3), with conserved disulfide bonds connecting the N-terminus to ECL3 and ECL1 to ECL2; the longest loop, ECL2, contains a β-hairpin structure. The cytoplasmic face of the receptor includes three additional connecting loops (intracellular loops, ICL1, 2, and 3) and the C-terminal region, which is truncated in most structures but is expected to contain an additional helix (helix 8) and the site of attachment for a lipid anchor.

Chemokines fold into a conserved, compact tertiary structure consisting of a 3-stranded antiparallel β-sheet packed against a single α-helix (Figure 3b). The ~20–25 residues preceding the first β-strand consist of: an unstructured N-terminal region (~10 residues), the conserved cysteine-containing motif (CC, CXC, CX3C or C), an irregularly structured loop designated the “N-loop”, and a single turn of 3_10_-helix. The first conserved cysteine residue forms a disulfide bond to the “30s loop”, located between the β1- and β2-strands, whereas the second conserved cysteine residue forms a disulfide bond to the β3-strand. Thus, the disulfides are essential for formation of the folded chemokine structure and receptor interactions.

The recent structures of chemokine-bound receptors [13,14], in addition to several structures of chemokines bound to receptor fragments [20,21,22], have confirmed two central aspects of the popular “two-site model” for chemokine-receptor interactions [23] (Figure 3c). First, the N-terminal region of the receptor binds to a shallow groove formed by the N-loop and β3-strand of the cognate chemokine. Second, although the chemokine N-terminus is disordered in the free chemokine, it binds to a site buried within the receptor transmembrane helical bundle, thereby undergoing induced fit to the receptor. The two-site model implies that these two aspects of the interaction occur sequentially as two separate steps, representing initial binding and subsequent activation. However, it has now become clear that elaborations of this model are necessary to explain many subtleties of the chemokine-receptor network [24]. Importantly, while structures of a few activated GPCRs have now been described [25], there is no structure for a chemokine-receptor complex in the activated state, so the structural basis of transmembrane signaling remains to be established. Nevertheless, a recent shotgun mutagenesis study of CXCR4 has identified a network of interactions likely to participate in signal transmission during receptor activation (Figure 3c) [19].

## 3. Genetic and mRNA Splice Variants of Chemokines and Receptors

### 3.1. Variation Between Species

Chemokine-receptor systems are present in all mammals, as well as some more primitive vertebrates [26,27], but have been most extensively characterized in humans and mice. Most human chemokines and receptors have orthologs in mice and vice versa, allowing mouse experiments to reveal biological functions relevant to human physiology and disease. However, some chemokines (CCL13/MCP-4, CXCL8/IL-8, CCL14/HCC-1, CCL18/DC-CK-1/PARC) are expressed in humans but not mice and, conversely, two chemokines (CCL12/MCP-5 and CXCL15/Lungkine) have been reported in mice but no ortholog has yet been identified in humans [28,29]. Similarly, mice express the chemokine receptor Ccr1-like1 (Ccr1l1), which is not found in humans [29]. Moreover, it should be noted that the selectivity of chemokine-receptor binding and activation, their expression patterns and other mechanisms of functional regulation, may differ between species, so extrapolating the conclusions of animal experiments to humans should be approached with care.

### 3.2. Polymorphisms in Chemokine Genes

A variety of polymorphisms have been identified in either the coding or non-coding regions of chemokine genes. These have the potential to alter expression levels, stability, and interactions with receptors or other binding partners. Consequently a number of these polymorphisms have been associated with increased or decreased disease progression.

Seven single nucleotide polymorphisms (SNPs) have been reported in the CCL2/MCP-1 gene. Four of these are present in the distal regulatory region, one in the promoter region, one in the first intron and one in the 3′ flanking region [30]. Four of these SNPs are associated with increased levels of MCP-1 protein expression [30]. In particular, the SNP −2578 A/G, in the distal regulatory region, increases the level of MCP-1 expression, occurs at higher frequency in individuals with complications of atherosclerosis such as myocardial infarction and stroke [30,31], and has also been associated with systemic sclerosis [32], multiple sclerosis [33], rheumatoid arthritis [34], and Alzheimer’s disease [35]. On the other hand, in a large cohort of HIV patients, homozygosity for the MCP-1 −2518 G allele was associated with a 50% reduction in the risk of acquiring HIV-1, although after infection this MCP-1 genotype enhanced disease progression with a 4.7-fold increased risk of HIV-related dementia [36].

Hellier et al. (2003) [37] have reported two polymorphisms in the promoter region of the CCL5/RANTES gene. The −403G→A polymorphism has been found to increase CCL5 expression, thus leading to increased sensitivity to asthma, atopy, and HIV [38]. In contrast, although the chemokine CCL17/TARC is thought to contribute to allergic disorders, the −431 C/T SNP in the gene encoding CCL17 increases chemokine expression without enhancing susceptibility to atopic dermatitis [38].

The chemokine CXCL12/SDF-1 is the ligand for the receptor CXCR4, which also acts as a coreceptor for HIV infection of leukocytes, especially T cells. A SNP has been reported in the 3′-untranslated evolutionarily conserved region of the gene encoding CXCL12 and homozygotes for this SDF1-3′A allele have shown a phenomenal protection against AIDS [39]. One hypothesis is that the polymorphism leads to overexpression of the chemokine thereby binding a high proportion of CXCR4 molecules and preventing their interaction with the viral coat protein [40].

Finally, Hellier et al. [37] have reported a polymorphism in the coding region of CCL8/MCP-2, causing a mutation of Gln-46 to Lys. Although the functional effect of this mutation is currently unknown [37], this mutation occurs in a region of the chemokine known to be involved in electrostatic attraction between the positively charged chemokine and negatively charged receptor, so we speculate that it may influence affinity and potency.

### 3.3. Polymorphisms in Chemokine Receptor Genes

As noted for chemokine ligands, a number of polymorphisms have also been identified in the genes encoding chemokine receptors. For example, in CCR2, nine SNPs have been observed that are associated with susceptibility to and severity of several diseases, including atherosclerosis, pulmonary disease, multiple sclerosis, HIV and hepatitis C virus infection, and cancer [41,42,43,44,45]. In contrast, the CCR2 polymorphism 190 G/A, which gives rise to a conservative amino acid change from valine to isoleucine in the first transmembrane helix of the receptor, is associated with delayed progression of HIV, apparently because it indirectly reduces the cell surface expression of the HIV-co-receptor CCR5 [46,47].

CCR5 is the co-receptor for infection of macrophages by M-tropic strains of HIV. A 32bp deletion in CCR5 to give the variant CCR5-∆32bp was first identified in 1996 [48,49]. This 32bp region codes for a region corresponding to the second extracellular loop of CCR5; the deletion causes a frame shift, leading to early termination of translation, resulting in a truncated non-functional protein, which lacks three trans-membrane segments of the receptor [50]. Thus, the CCR5-∆32bp mutation provides strong protection against HIV-transmission and causes a delay in disease progression [51].

The Duffy antigen receptor for chemokines (DARC) was identified first as the human blood group antigen, but was later determined to be an atypical chemokine receptor (see below) and the cell-surface protein used by the malarial parasite *Plasmodium vivax* to invade red blood cells. A polymorphism (−46C) in the promoter region of the *DARC* gene, if homozygous, disturbs the binding site for the transcription factor GATA-1, thereby reducing DARC expression and yielding protection against *P. vivax* infection [52].

### 3.4. Chemokine Receptor Splice Variants

In addition to genetically encoded mutations, the amino acid sequences of chemokine receptors can be influenced by alternative splicing of precursor mRNA. For example, CCR2 can exist as two splice variants, CCR2A (360 amino acids) and CCR2B (374 amino acids), which differ from each other in their carboxy terminal regions [53]. Bartoli et al. [54] have shown that these isoforms are expressed in different cell types in idiopathic inflammatory myopathies. As another example, CXCR3, which is found to be involved in cancer metastasis and inflammatory diseases, exists in three differentially spliced forms—CXCR3A, CXCR3B, and CXCR3Alt. CXCR3A and CXCR3B differ only in the lengths of their N-terminal regions; CXCR3B has a longer N-terminus containing two additional potential sulfation sites (see below). However, the third variant CXCR3Alt is a truncated protein working more like an atypical or decoy receptor. It has five transmembrane helices, with a short C-terminal region and lacks the third intracellular loop [55]. These splice variants have been reported to show specific expression in particular cell types leading to different functional characteristics. Recently, it has been shown that these variants activate different signaling pathways and show tissue-specific biased agonism [55].

## 4. Regulation of Expression, Degradation and Localization

### 4.1. Expression

Some chemokines and receptors are constitutively expressed in specific tissues and cell types, contributing to homeostatic functions such as T cell development, stem cell migration, and lymphoid organogenesis [56]. Others are induced at sites of injury or infection as part of the inflammatory response. Moreover, a few chemokines and their receptors appear to have both homeostatic and pro-inflammatory functions. Detailed classifications of the homeostatic versus inflammatory chemokines and receptors have been presented previously [29,57,58].

Homeostatic chemokines and receptors tend to be specific for each other, as exemplified by the chemokine CXCL12 and its receptor CXCR4. CXCL12, the only cognate chemokine of CXCR4, is constitutively expressed by bone marrow stromal cells, whereas CXCR4 is expressed on hematopoietic stem cells. Thus, the activation of CXCR4 by CXCL12 promotes homing of hematopoietic stem cells to the bone marrow [59]. Plerixafor (AMD3100), one of the two commercialized chemokine receptor antagonists, can inhibit the interaction between CXCL12 and CXCR4, thereby enhancing the mobilization of hematopoietic stem cells into peripheral blood for stem cell collection and transplantation [60]. A second homeostatic chemokine-receptor pair is CCL25/TECK, which is constitutively strongly expressed in the thymus with relatively low expression in other organs [61], and CCR9, expressed on T cells. Similarly, the homing chemokine CCL19/MIP-3β/exodus-3 is highly expressed in the T cell zone of lymph nodes where it plays a role in T cell recruitment and migration by activation of the receptor CCR7 [62,63].

In contrast to the few homeostatic chemokines and receptors, the majority of chemokines and receptors are upregulated in response to inflammatory stimuli. Indeed there are hundreds (perhaps thousands) of papers reporting increased chemokine and/or receptor levels in diseased tissues compared to healthy controls. Moreover, often numerous chemokines and/or receptors are highly expressed in the same disease tissue, making it difficult to separate the causes and effects of the inflammation. As one example, Iwamoto and co-workers have described the increased expression of six CC chemokines, five CXC chemokines, XCL1, and CX3CL1 in the synovial tissues of rheumatoid [64] arthritis patients, compared to other forms of arthritis or healthy controls [65]. The consequent infiltration of leukocytes into the joints is thought to contribute to fibrosis and cartilage and bone degradation. Overexpression of chemokines is also a common feature of tumors. For example, the expression of CCL5 was increased more than 50-fold in primary breast cancer tissue compared to the normal tissue adjacent to the tumor [66,67,68].

### 4.2. Internalization and Recycling or Degradation

After binding and activation by chemokines, chemokine receptors typically undergo internalization, followed by either degradation or recycling to the plasma membrane. The well-studied mechanism of receptor internalization is clathrin-mediated endocytosis [64]. The process starts with receptor activation by the ligand and phosphorylation (mediated by G protein receptor kinases, GRKs) of serine or threonine residues near the C-terminus of the receptor, leading to receptor desensitization. The phosphorylated receptors, containing the “dileucine” motif, facilitate the recruitment of endocytosis-related molecules adaptin 2 (AP2) and β-arrestin [69]. The complex of the receptor with AP2 and β-arrestin further attracts clathrin, leading to internalization of the receptor from the plasma membrane to form clathrin-coated vesicles. The receptor and ligand are then unloaded to endosomes in which the chemokine and receptor can dissociate under the acidic endosomal conditions allowing the receptor to be recycled back to the cell membrane [69]. Alternatively the receptor and ligand can be transported to the lysosome for degradation. Studies of receptors CCR5 and CXCR2 suggest that a PDZ ligand domain at the C-terminus of receptors can direct sorting of the receptors between recycling or degradation pathways [70,71].

There is considerable variability in the susceptibility of different chemokine receptors to lysosomal degradation. Whereas some receptors, such as CCR5 or CCR7, are resistant to degradation in the lysosome, activated CXCR4 undergoes lysosomal degradation through ubiquitination at a lysine residue in the C-terminus by E3 ubiquitin ligase AIP4 [64,72,73]. On the other hand, CXCR7 and CCR7 can be constitutively ubiquitinated. Ligand activation can lead to the de-ubiquitination of CXCR7, resulting in receptor recycling, whereas ligand-induced de-ubiquitination was not observed for CCR7 [74,75]. Further investigation is required to better define the determinants of receptor ubiquitination and degradation.

### 4.3. Atypical (Decoy) Chemokine Receptors as Chemokine Scavengers

Although the major function of chemokine receptors is to guide the migration of leukocytes in response to chemokines, there are several receptors that can recognize chemokines without eliciting the classical GPCR signaling events or chemotaxis. These receptors, variously referred to as “decoy” or “silent” chemokine receptors, have now been classified as the “atypical” chemokine receptor family (ACKR). The family consists of four major receptors (ACKR1/DARC, ACKR2/D6, ACKR3/CXCR7, and ACKR4/CCX-CKR/CCRL1) with two others (ACKR5/CCRL2 and ACKR6/PITPNM3) under further functional investigation.

The atypical receptors share the seven transmembrane helix domain of chemokine receptors and bind to chemokines, but most of them lack the highly conserved “DRYLAIV” motif in the second intracellular loop, which is involved in G protein activation. Rather than inducing G protein-mediated signals, atypical receptors are able to signal by recruitment of β-arrestin and consequent internalization and degradation of the receptor and bound chemokine. Notably, atypical chemokine receptors tend to display high ligand promiscuity (Figure 2). Thus, the main function of ACKRs is thought to be regulation of the innate and adaptive immune response by acting as a chemokine reservoir or scavenger [76,77,78,79,80,81]. This role has been demonstrated both in vitro and in vivo for ACKR2/D6, the first receptor to be functionally classified as an atypical chemokine receptor [82,83]. Interestingly, in addition to being an atypical chemokine receptor ACKR1/DARC (Duffy Antigen and Receptor for Chemokines) is the human blood group antigen and is also the receptor for infection of reticulocytes by the malarial parasite *Plasmodium vivax* [8].

### 4.4. Localization by Binding to Glycosaminoglycans (GAGs)

Chemokines, particularly in their oligomeric forms (see below), bind avidly to glycosaminoglycans (GAGs), polysaccharides expressed on the surfaces of most cells. Based on their repeating disaccharide units, GAGs can be divided into four groups: heparin/heparan sulfate, chondroitin sulfate/dermatan sulfate, keratan sulfate, and hyaluronic acid [84]. The highly sulfated and acidic GAGs bind to basic residues within chemokines through electrostatic interactions. These interactions are thought to help maintain high local concentrations of chemokines near their sites of expression (inflammatory loci), to establish concentration gradients of chemokines that promote leukocyte chemotaxis, and possibly to directly present chemokines to their receptors [85]. The importance of chemokines binding to GAGs has been convincingly demonstrated in vivo; chemokine mutants defective in GAG binding but capable of receptor activation in vitro are severely impaired in their ability to induce leukocyte chemotaxis in animal experiments [86]. Moreover, the effects of such mutations may be tissue-specific. For example, mutation of GAG-binding residues reduces the neutrophil recruitment activity of CXCL8 in the peritoneum but enhances this activity in the lung [87].

In addition to simple localization of chemokines, GAGs may regulate chemokine function by indirect mechanisms. Different cell types (or diseased versus healthy cells) express different arrays of GAGs, which can selectively bind to different chemokines. For example, CCL5 has high affinity for both heparin and dermatan sulfate, whereas CCL2 can bind more strongly to heparin or heparan sulfate than to chondroitin sulfate or dermatan sulfate [88]. Thus, GAGs could influence the relative concentrations of available chemokines and thereby the populations of different leukocytes recruited. Finally, as discussed below, by selectively binding to chemokine oligomers GAGs also promote chemokine oligomerization, which could, in turn, influence their receptor activation and protect them from proteolysis [84].

## 5. Post-Translational Modifications

### 5.1. Proteolytic Processing of Chemokines

Chemokines are initially translated with a ~23 amino acid signal sequence, which is cleaved prior to secretion of the mature protein. However, careful biochemical analysis of chemokines isolated from biological samples has shown that they may be further processed by either N-terminal or C-terminal truncation; often both full-length and one or more truncated forms are observed [89,90]. Truncation occurs through the catalytic action of proteases, some of which have been identified [90]. In particular, matrix metalloproteinases can process each of the monocyte-directed CC chemokines near their N- or C-termini [91].

Structure–function studies have highlighted a crucial role for the N-terminal regions of chemokines in receptor activation. Consistent with these observations, natural N-terminal truncation can either increase or decrease the activity of chemokines at their receptors or can alter their selectivity across several receptors. For example, the neutrophil chemoattractant CXCL8 exists in two forms (−2–77 and 1–77) resulting from alternative signal peptide cleavage [92]. These two proteins have different susceptibility to subsequent cleavage by aminopeptidases, giving rise to two additional forms (2–77 and 3–77), which have enhanced affinity for heparin. Moreover, further proteolytic processing catalyzed by the blood coagulation proteases thrombin or plasmin gives a shorter form (6–77) with increased chemotactic activity. Interestingly, CXCL8(6–77) is also formed under the action of a bacterial protease in cultures of the periodontal pathogen *Porphyromonas gingivalis*, apparently a mechanism to elicit an enhanced host response to this pathogen [93]. The complexity of chemokine N-terminal truncation is further demonstrated by the case of CCL14. Full-length CCL14(1–74) is a weak CCR1 agonist but is cleaved by plasmin or urokinase plasminogen activator to give CCL14(9–74), a potent agonist of CCR1 [94]. However, subsequent removal of two additional residues, catalyzed by dipeptidyl peptidase IV (CD26), gives biologically inactive CCL14(11–74) [95]. Interestingly, the most active form, CCL14(9–74), is also most efficiently bound, internalized and degraded by the decoy receptor D6 [96], suggesting that D6 internalization and dipeptidyl peptidase IV cleavage represent two alternative strategies to accomplish the same biological effect.

Truncation of chemokines at their C-termini is much less likely to influence receptor activation as this region of chemokines does not directly interact with chemokine receptors. However, the C-terminal region may be involved in oligomerization and/or GAG binding. Thus, for example, the splice variant of CXCL12 known as SDF-1α undergoes removal of a single C-terminal residue in human serum, thereby reducing its ability to bind to heparin or to cell surfaces and to stimulate cell proliferation and chemotaxis, although the truncation has no effect on receptor activation in vitro [97]. On the other hand, the chemokines MIP-1α and MIP-1β, are cleaved internally ~8–11 residues from their C-termini and then further degraded, thus inactivating these chemokines [98]. Cleavage of these chemokines by the protease cathepsin D is highly selective over other chemokines and is suggested to play a role in reducing the immune response to breast tumors [98].

### 5.2. Other Post-Translational Modifications of Chemokines

In addition to proteolytic processing, a number of other chemokine post-translational modifications have been observed. *N*- and *O*-glycosylation are common modifications of secreted proteins. *O*-glycosylation has been observed on several chemokines, including CCL2, CCL5, CCL11/eotaxin-1, CCL14, and CX3CL1/fractalkine [99,100,101,102,103,104]. Although glycosylation has not been shown to directly influence receptor activation by these chemokines, both CCL5 and CCL14 are glycosylated near to their N-termini [102,103] so it seems likely that receptor interactions will be affected. In addition, it is possible that glycosylation may have indirect effects on chemokine function by altering stability, rates of clearance, or localization.

**Figure 4:**
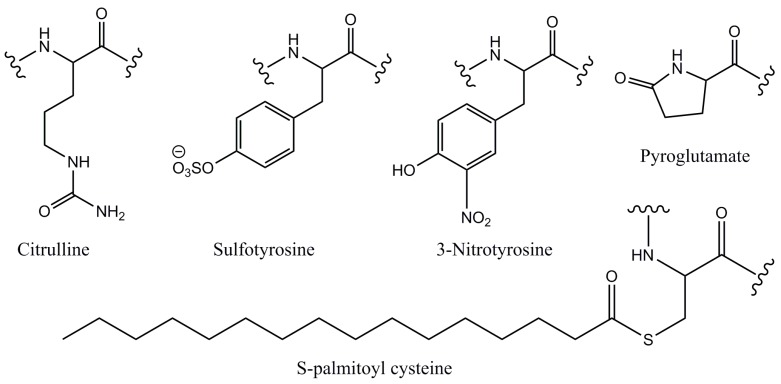
Examples of post-translationally modified amino acid residues found in chemokines and chemokine receptors.

Pyroglutamate is a modified amino acid formed by the dehydration and cyclization of an N-terminal glutamine or glutamate residue in a peptide or protein (Figure 4). This transformation is catalyzed by the enzyme glutaminyl cyclase in vivo but can also occur spontaneously in vitro [105]. Human and some murine chemokines of the monocyte chemoattractant protein family (CCL2, CCL8, CCL7/MCP-3, and CCL13) have N-terminal glutamine residues that undergo pyroglutamate formation [106,107,108]. There are conflicting reports regarding the influence of this modification on receptor activation by CCL2 [109,110]. However, pyroglutamate has been found to increase the stability of CCL2 to N-terminal degradation by aminopeptidases [109]. Moreover, glutaminyl cyclase contributes to the pathogenesis of both Alzheimer’s disease and fatty liver disease and pyroglutamate formation of CCL2 has been suggested as a possible underlying mechanism in both diseases [111].

Citrullination is the deimination of arginine residues of peptides or proteins to yield citrulline residues (Figure 4). This modification is catalyzed by enzymes called peptidylarginine deiminases and is known to occur to several chemokines influencing their activities [112,113]. CXCL8 undergoes citrullination at Arg-5, resulting in slightly higher affinity for CXCR1 and slightly lower affinity for both CXCR2 and heparin in vitro [113]. Curiously, in vivo models gave contrasting results, with citrullinated CXCL8 being less effective at inducing neutrophil extravasation into the peritoneal cavity recruitment [113] but more effective at mobilizing neutrophils from the bone marrow into the bloodstream [114]. For other chemokines studied—CXCL5/ENA-78, CXCL10/IP-10, CXCL11/I-TAC, and CXCL12—citrullination generally decreased receptor binding or activation [115,116,117].

One common feature of inflamed tissues is oxidative stress, the production of reactive oxygen and nitrogen species, which can subsequently modify proteins, influencing many biochemical functions. In particular, some chemokines have been shown to undergo nitration, which typically occurs on tyrosine or tryptophan side chains, as a consequence of reaction with peroxynitrite (Figure 4) [112]. CCL2 underdoes nitration in response to macrophage activation [118] and nitrated CCL2 has reduced monocyte-binding and chemotactic function [118,119]. Similarly nitration of CCL5 attenuates its chemotactic activity [120]. In addition to direct modification of chemokine structure and activity, nitration or nitrosylation (addition of NO rather than NO_2_) of other proteins can indirectly influence chemokine or receptor function. For example, nitrosylation of mitogen-activated protein kinase phosphatase 7 deactivates this phosphatase, thereby enabling signaling in response to the chemokine CXCL12 [121] and nitric oxide synthesis can promote expression of the receptor CXCR4 [122].

### 5.3. Post-Translational Modifications of Chemokine Receptors

Chemokine receptors are also subject to a variety of post-translational modifications, with implications for chemokine recognition and signaling. As is typical for GPCRs, chemokine receptors are reversibly phosphorylated on their cytoplasmic regions, thereby desensitizing them to activation and regulating binding by β-arrestins and consequent internalization [69,123,124,125]. Palmitoylation (Figure 4) and ubiquitination have also been suggested to influence these receptor functions, as discussed previously [69]. Here, we focus on the two most widely studied modifications of chemokine receptors, glycosylation, and tyrosine sulfation.

Many (or perhaps most) chemokine receptors, including decoy receptors and viral receptor mimics, are heterogeneously *N*- and/or *O*-glycosylated, in some cases influencing receptor function [126,127,128,129,130,131,132,133,134,135]. For example, removal of sialic acid moieties from CCR5 significantly reduced the efficacy of signaling by chemokines at this receptor but had little effect on CCR5-mediated HIV-1 infection [134]. On the other hand, *N*-linked glycosylation was found to influence the ability of CXCR4 to support infection by different strains of HIV-1 [133]. Similar to CCR5, CCR7 is polysialylated in its extracellular domain and this modification is required for recognition of the chemokine CCL21/6Ckine/SLC/exodus-2 [136,137]. The importance of this modification in vivo was demonstrated using polysialyltransferase-deficient mice, which lacked the ability to recruit dendritic cells to secondary lymphoid organs in response to inflammatory challenge [137]. Moreover, human dendritic cells were found to further regulate CCR7 activity by secreting enzymes that deglycosylate this receptor [136]. As noted for chemokines, receptor glycosylation can also have secondary effects such as protection from proteolysis, which was observed for CXCR2 [138]. Finally, in a fascinating response to bacterial infection, proteases in the airways of cystic fibrosis patients were found to cleave CXCR1 expressed on neutrophils, releasing glycosylated CXCR1 peptides [139]. These peptides then acted on Toll-like receptors on bronchial epithelial cells to stimulate expression of CXCL8, a major ligand for CXCR1 and CXCR2, apparently a mechanism to promote additional neutrophil recruitment.

Another important post-translational modification of chemokine receptors is tyrosine sulfation, addition of a sulfate group, and a negative charge to the phenolic hydroxyl of a tyrosine side chain (Figure 4). Sulfation is a common modification of secreted proteins mediated by two tyrosylprotein sulfotransferase enzymes, which are localized to the trans-Golgi network [140]. These enzymes are selective for tyrosine residues close in sequence to acidic amino acids, a motif found in the N-terminal (chemokine-binding) regions of most chemokine receptors [141]. Several chemokine receptors have been shown to be sulfated [8,135,142,143]. Mutation of the sulfated residues, or metabolic inhibition of sulfation, tends to reduce chemokine binding affinity and potency of receptor activation [141]. Moreover, many chemokine receptors contain two or more tyrosine residues in their N-terminal regions and the pattern of sulfation could potentially modulate selectivity for different ligands. In support of this mechanism, Choe et al. [8] have found that mutation of Tyr-41 in the decoy receptor DARC suppresses binding of CCL2, CCL5, and CXCL1/GROα/MGSA-α but not CXCL8, whereas mutation of Tyr-30 of DARC suppresses binding of CXCL8 but not the other three chemokines.

We and others have used tyrosine-sulfated peptides derived from the N-terminal regions of chemokine receptors to understand the structural and energetic basis of chemokine recognition [144]. These studies have confirmed that sulfation enhances affinity and selectivity of chemokine binding [145,146,147,148] and have also suggested that binding to the sulfated region of the receptor can allosterically modulate chemokine dimerization [147,148,149]. Moreover, NMR structural studies using these model systems have shown that the sulfotyrosine residues bind into an electropositive groove on the chemokine surface, with distinct orientations for different chemokine-receptor pairs [21,22]. These structures provide insights into the mechanism by which sulfation regulates receptor function.

## 6. Oligomerization of Chemokines and Chemokine Receptors

### 6.1. Oligomerization of Chemokines

**Figure 5:**
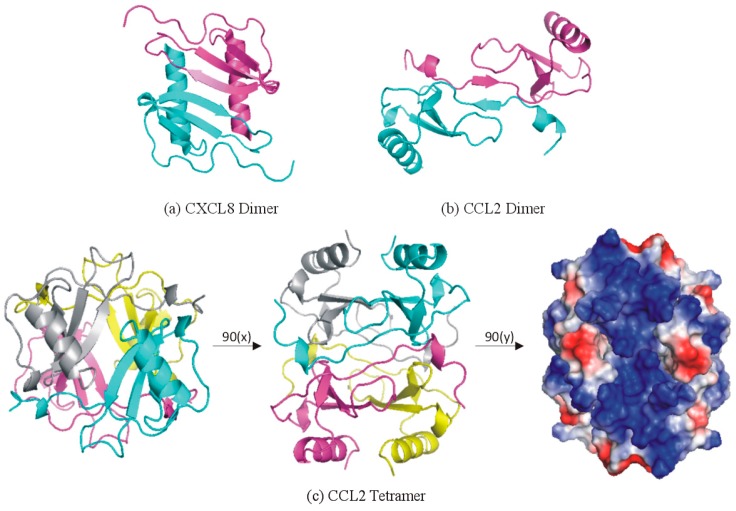
Oligomeric structures of chemokines. (**a**,**b**) Dimer structures of (**a**) CXCL8/IL-8 and (**b**) CCL2/MCP-1, highlighting the distinct dimer interfaces for CXC and CC chemokines, respectively. (**c**) Tetramer structure of CCL2, highlighting: (left) the CXC-type dimer interfaces (cyan to gray and magenta to yellow protomers); (center) the CC-type dimer interfaces (cyan to magenta and yellow to gray protomers); and (right) the highly electropositive (dark blue) surface involved in GAG binding.

**Figure 6:**
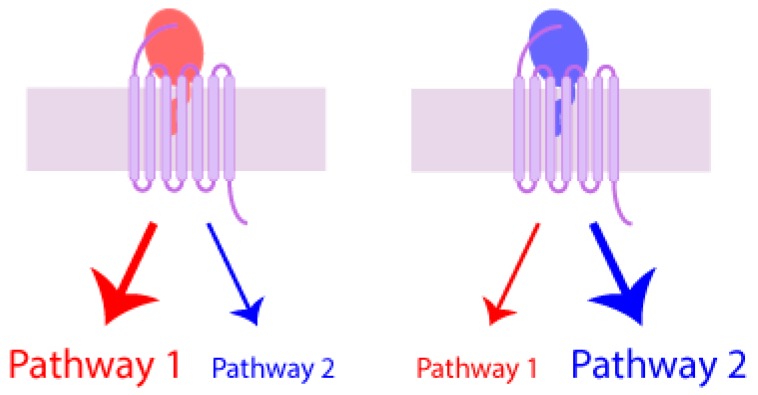
Schematic representation of biased agonism. The red chemokine (**left**) selectively activates pathway 1, whereas the blue chemokine (**right**) selectively activates pathway 2.

Most chemokines form dimeric or higher order oligomeric structures [84]. Strikingly, the CXC and CC chemokines generally form distinct dimer structures (Figure 5). CXC chemokines dimerize via their β1-strands, thereby forming a continuous 6-strand antiparallel β-sheet with the α-helices of both protomers adjacent to each other on the same face of the β-sheet (Figure 6). Importantly, this dimer structure leaves the N-terminus, N-loop and β3-strand exposed on the surface of the dimer. Therefore, CXC chemokine dimers can bind and activate chemokine receptors [150,151,152,153]. Indeed, trapped forms of the CXCL8 and CXCL12 dimers (i.e., those that cannot dissociate to the monomeric state) displayed distinct receptor activation properties relative to corresponding monomeric chemokines [151,152,154]. In contrast to CXC chemokines, CC chemokines dimerize by formation of an antiparallel β-sheet between the N-terminal regions of the two protomers (Figure 5b). Due to the importance of the N-terminal regions in receptor activation, CC chemokine dimers are inactive [155,156].

In some cases, chemokine dimers can further associate to form tetrameric structures, containing both CXC- and CC-type dimers (Figure 5c) [18,157], or to form high order aggregates [158,159]. Due to the burial of N-terminal and N-loop elements, these higher order structures are also expected to be unable to activate receptors.

Chemokine dimers can readily dissociate into the monomeric forms. Considering that the equilibrium dissociation constants for dimerization are generally in the micromolar to millimolar regime and the physiological concentrations of chemokines are generally expected to be sub-micromolar, oligomerization was initially considered to be a possible artefact of the high concentrations used for structure determination. However, it is now apparent that oligomerization is critical for binding to GAGs and thereby creating the localized chemokine gradients required for effective chemotaxis [84,85,86,160,161,162]. Figure 5c highlights the electropositive surface that forms upon tetramerization of CCL2, thereby promoting cooperative binding to GAG polymers.

Most studies of chemokine dimers have utilized conditions under which a single chemokine is present or dominant. However, physiological circumstances more often involve multiple chemokines being present, raising the possibility that they could form heterodimers (or higher oligomers) and adding a further level of complexity to structural regulation. In support of this possibility, Mayo and coworkers have shown that CXCL4/PF4 and CXCL8 form heterodimers and that the heterodimers have enhanced anti-proliferative and chemotactic activity in comparison to homo-oligomers of either chemokine [163]. Detailed molecular dynamics calculations further predict the formation of heterodimers between other pairs of CXC chemokines, between various CC chemokines and between CXC and CC chemokines [164,165]. Considering that many chemokines exist in multiple post-translationally modified forms, it is also likely that heterodimers can assemble from different forms of the same chemokine. In support of this possibility, a synthetic, N-terminally truncated, inactive form of CCL2 can bind to full-length CCL2, thereby competitively inhibiting receptor activation [166].

Finally, Volkman and colleagues have characterized a remarkable structural transformation of the only XC chemokine XCL1/lymphotactin [167,168]. Monomeric XCL1 has the canonical chemokine fold, is an agonist of receptor XCR1 and does not bind appreciably to GAGS. However, homodimeric XCL1 has a completely different tertiary and quaternary structure from those of other chemokines and is inactive at XCR1 yet binds with high avidity to GAGs. Although interconversion of the two structural forms requires unfolding and refolding of the protein, the two states can interconvert rapidly. Moreover, both states are significantly populated under typical physiological conditions but the equilibrium between them is sensitive to solution conditions such as salt concentration and temperature and is regulated by GAG binding.

### 6.2. Oligomerization of Chemokine Receptors

A further level of complexity in chemokine-receptor interactions is oligomerization of receptors. GPCRs are generally thought to exist as dimers or possibly higher order oligomeric complexes and most chemokine receptor structures determined to date have been homodimers. Chemokine receptors could potentially self-associate (to form “homomers”), associate with other chemokine receptors (to form “heteromers”), or associate with other non-chemokine GPCRs. Examples of all three have been reported. Moreover, the formation of such oligomers could potentially influence receptor function by a variety of mechanisms, including: modulating interactions with chemokine ligands; modulating interactions with signaling effectors such as G proteins; affecting trafficking to the plasma membrane; affecting localization within the membrane; altering receptor stability; or modulating internalization and/or receptor recycling to the membrane. Many of these potential effects have been investigated for chemokine receptors, as discussed in several detailed reviews [161,169,170,171,172].

Chemokine receptors, like other GPCRs, dimerize by approximately parallel association of their transmembrane (TM) helices, although the specific helices involved in the interactions may vary. In crystal structures CXCR4 dimerizes by association of TM4 and TM5 [14,15], whereas CCR5 (in complex with the drug maraviroc) dimerizes by association of TM1 and TM7 [17], although mutational analysis of CCR5 suggested that dimerization involves residues in TM1 and TM4 [173]. Irrespective of the specific TM helices involved in dimerization, it seems reasonable to expect that ligand binding within the TM bundle may affect not only the conformation of the bundle (as required for signaling) but also the structure and/or stability of the dimerization interface. Conversely, formation of different dimers may affect ligand binding.

Evidence for homo- and/or hetero-oligomerization in cells has been reported for numerous CC and CXC chemokine receptors and for the decoy receptor DARC [169]. Generally, these receptor oligomers have been found to exist constitutively, i.e., not to require induction by ligand binding or activation. However, in many cases, activation by chemokines induces a change in the oligomer structure or appears to increase homomer formation [169]. It should be noted that observation of oligomer formation in cells typically requires heterologous expression of receptors modified with tags to facilitate detection by antibodies (typically using co-immunoprecipitation experiments) or by fluorescence or bioluminescence resonance energy transfer (FRET or BRET) techniques. Changes in the signals observed in these experiments may result either from alterations in the populations of multimeric species or from conformational changes within preformed oligomers. Therefore, the results should be interpreted with care. Nevertheless, the extensive body of experimental evidence provides a high level of confidence that chemokine receptors do oligomerize and that the oligomers are sensitive to ligand binding.

The interplay between oligomerization and ligand binding is exemplified by the observations that different ligands can induce distinct alterations in receptor oligomer structure. For example, the chemokines CCL2 and CXCL12 differentially influenced the conformations (measured as maximal BRET signals) of CXCR4 homomers and CCR2/CXCR4 heteromers without affecting the propensity of these oligomers to assemble [174]. Coupling between ligand binding and dimerization has also been inferred by comparing competitive ligand binding measurements for receptors expressed alone or together with a receptor with which it forms heteromers. Thus, for example, the CCR5 ligand CCL4/MIP-1β does not bind directly to CCR2 and therefore cannot displace the cognate chemokine CCL2 from CCR2 when this receptor is expressed alone. However, when CCR2 is co-expressed (and dimerized) with CCR5, CCL4 effectively displaces CCL2 from binding to CCR2 within the heteromer [175]. These data suggested that there can be strong negative allostery for chemokine binding to receptor protomers within a dimer or, taken to its extreme, that only one ligand can bind to a receptor dimer.

A critical question regarding receptor oligomers is whether they simply act as the sum of the two contributing receptors or alternatively synergize to yield more sensitive or different downstream signaling responses. In a seminal study, Mellado et al. [176] have reported that cells co-expressing CCR2 and CCR5 respond cooperatively to ligands of these receptors. Specifically, simultaneous treatment with CCL2 and CCL5 (ligands for CCR2 and CCR5, respectively) induced a Ca^2+^ signal equivalent to treatment with an approximately 10-fold higher concentration of each individual chemokine. The allosteric response to the combination of ligands was supported by cross-linking experiments showing that simultaneous treatment with CCL2 and CCL5 either induced heteromer formation or altered the conformation of existing heteromers. Mellado et al. further examined the effect of the dominant negative CCR2 mutant CCR2B Y13F on cooperative signaling. Remarkably, this mutant was shown to heterodimerize with CCR5 such that CCL5 signaling was inhibited by simultaneous treatment with CCL2. Further experiments indicated that effective signaling via the CCR2-CCR5 heteromer appears to require both receptors to be signaling competent and to form distinct complexes with downstream kinases. Finally, the same study revealed that the Ca^2+^ signal induced by simultaneous treatment with CCL2 and CCL5 was not sensitive to the G_αi_ inhibitor pertussis toxin, whereas the Ca^2+^ signals induced by the individual chemokines were inhibited by pertussis toxin. Thus, allosteric signaling at the CCR2-CCR5 heteromer induced a unique downstream signaling pathway in comparison to each individual receptor.

As discussed above, one important aspect of chemokine-receptor regulation is the ability of ligands to induce internalization of their receptors and to be internalized themselves in the process. The decoy receptor DARC utilizes this mechanism to act as a chemokine scavenger. However, it has also been shown that DARC can form heteromers with CCR5 that block CCR5 signaling in response to its ligands but still permit ligand binding and internalization of CCR5 [177]. Thus, DARC reduces the local concentrations of chemokines not only by direct internalization but also by promoting chemokine internalization indirectly via oligomerization with other receptors.

In addition to oligomerizing with themselves or each other, some chemokine receptors have also been found to form heteromers with other members of the GPCR superfamily. In one example, CCR5 has been shown to form heteromers with the complement C5a receptor and C5a-stimulation of this receptor caused cross-phosphorylation as well as internalization of CCR5 [123]. In a second example, Mustafa et al. [178] showed that CXCR2 forms heteromers with the α_1A_-adrenoceptor (α_1A_AR), that the α_1A_AR agonist norepinephrine stimulated recruitment of β-arrestin only in cells co-expressing CXCR2, and that norepinephrine-stimulated β-arrestin recruitment could be inhibited by an allosteric inverse agonist of CXCR2. Finally, several studies have provided evidence for heteromer formation and allosteric regulation between chemokine receptors CCR5, CXCR2, and CXCR4 and various members of the opioid receptor family [179,180,181,182]. Considering the vast number of GPCRs and their co-expression in many of the same cell types as chemokine receptors, it is likely that our current knowledge of heteromer formation and their functional consequences barely scratches the surface.

## 7. Regulation of Signaling Pathways

### 7.1. Overview of Signaling Pathways—G Proteins and Arrestins

Chemokine receptors are members of the GPCR superfamily, the largest class of membrane receptors in the human proteome. Chemokine binding induces a conformational change in the 7-transmembrane helix domain of the receptor, which can induce a variety of downstream signaling events mediated by either heterotrimeric G proteins or arrestins. These signals typically involve activation of other intracellular effectors such as adenylyl cyclase. The specific signals induced can be regulated by variations in the available signaling machinery in different cellular contexts as well as by differences between the intrinsic structural interactions of chemokine ligands with their receptor.

Upon activation, GPCRs function as guanine nucleotide exchange factors (GEFs), which allows the α subunit of the heterotrimeric G protein to transition from inactive (GDP-bound) to active (GTP-bound) and to dissociate from the βγ subunits. Both parts of the G protein are able to interact with other effectors to generate signal transduction. The Gα proteins are divided into four major classes based on their sequence and function: Gα_q_ activates phospholipase C to upregulate the level of intracellular calcium; Gα_s_ stimulates the production of cAMP; Gα_i_ inhibits the production of cAMP; and Gα_o_ controls other signaling functions [183,184]. The Gβγ dimer can act as a Gα inhibitor when bound to a Gα subunit, because it favors the interaction between Gα and GDP. However, when the Gβγ complex is dissociated from Gα, it can also participate in the signaling cascade. For example, Gβγ can regulate ion channels [185] and is also involved in phosphorylation of the extracellular signal-regulated kinases (ERK 1/2) via the protein kinase C/protein kinase A pathway [186,187].

The arrestin family also includes four subtypes. Arrestin-1 (visual arrestin) and arrestin-4 (cone arrestin) are located exclusively in retinal rods and cones. Arrestin-2 (or β-arrestin 1) and arrestin-3 (or β-arrestin 2) are non-visual arrestins that are expressed in numerous cell types. The affinity of β-arrestins for the non-phosphorylated (inactive) receptor is low which limits any basal activity. When the receptor is activated by an agonist, β-arrestin is able to displace the G protein before it is activated giving rise to G protein-independent β-arrestin signaling. On the other hand, when β-arrestin competes with the G protein after its activation, this leads to G protein-dependent β-arrestin signaling. The latter signaling has been well studied and include ERK phosphorylation, receptor internalization, and desensitization (see above) [188]. G protein-independent β-arrestin mediated signaling is less well characterized, but it has been identified using GPCR mutants unable to bind to G proteins, which are still able to signal through the ERK phosphorylation pathway [189,190].

### 7.2. Regulation of Signaling in Different Cellular Contexts

Regulation of chemokine receptor (and other GPCR) signaling pathways is a highly complex phenomenon that has to be considered with respect to its cellular context. There are approximately 20 Gα subunits, 5 Gβ subunits, and 12 Gγ subunits [184], resulting in a huge array of possible heterotrimeric G proteins. The expression levels of these various subunits vary between cell types or under different conditions and the different complexes are expected to compete with each other for association with a particular receptor. In addition, the availability of particular G proteins may depend on the presence of other GPCRs in the same cell. Moreover, the ability of a chemokine receptor to signal is also dependent on the presence of other factors such as regulators of G protein signaling (RGS) proteins, which negatively regulate G protein signaling by acceleration of GTP hydrolysis by Gα [191,192,193], or lipids, such as cholesterol, which can potentially influence receptor oligomerization and conformational changes [194,195].

Considering these potential variations in signaling pathways between cells, perhaps it should not be surprising that the literature describing chemokine-chemokine receptor signaling is full of apparent inconsistencies. As one example, different studies have concluded that CCL11 is a partial agonist and an antagonist of CCR2 [196,197,198]. Thus, although detailed mechanistic studies of chemokine-receptor interactions typically require carefully controlled experimental conditions using immortalized cell lines, it is important to validate the biological relevance of results using primary cells.

### 7.3. Partial Agonism

Within a specific cellular context, it is sometimes observed that different chemokines can induce different signals via the same receptor. The simplest type of differential signaling is partial agonism, in which one chemokine (defined as a “full agonist”) induces a maximal response whereas another chemokine (a “partial agonist”) induces a lower response than the full agonist, even when added at concentrations sufficient to fully saturate the receptor [199]. For example, Berchiche et al. [200] have reported that CCL2 activates CCR2 to induce maximal β-arrestin recruitment, whereas other chemokines induce a lower level of β-arrestin recruitment by activation of CCR2 in the same cell line. It is important to note that partial agonism is most readily detected using non-amplified (proximal) signaling assays such as β-arrestin recruitment or direct measurements of G protein dissociation. For more highly amplified assays (e.g., cAMP levels, Ca^2+^ levels or phosphorylation of downstream effectors), even partial activation of a receptor can give rise to maximal responses. In the latter case, a partial agonist is expected to have lower potency that a full agonist (i.e., a higher concentration will be require to attain the full signal), even if it associates with the receptor with the same affinity as the full agonist. Recently, we have observed this phenomenon for activation of CCR2 by CCL2, CCL7, and CCL8 (unpublished results).

### 7.4. Biased Agonism

Biased agonism, also known as functional selectivity or agonist-selective signaling, is an increasingly developed concept based on the idea that agonists acting at the same receptor can have different abilities to activate different signaling pathways, as shown schematically in Figure 6. This phenomenon has been observed for a variety of GPCRs and is believed to be due, at least in part, to the ability of receptors to adopt multiple active conformations, each leading to a different balance of signaling outcomes and each differentially stabilized by different ligands [201]. The phenomenon is of particular interest when designing therapeutic agents [202]. For example, if several agonists of one receptor have been selected for a desired function, it might be possible to minimize undesirable side effects by testing for other functions where the agonists may differ in their responses [203]. In order to better understand biased agonism and guide drug development, several models have been proposed to quantify agonist bias [204,205,206].

A number of chemokine receptors have been observed to display biased agonism. For example, the chemokines CCL19 and CCL21 both activate CCR7 to induce G protein activation and calcium mobilization but only CCL19 gives rise to desensitization of CCR7, which is mediated by β-arrestin recruitment [207]. In a systematic study of G protein versus β-arrestin bias for three CC and three CXC chemokine receptors, Rajagopal et al. [208] found significant levels of signaling bias for CCR1, CCR10, and CXCR3. Similarly, Corbisier et al. [209] found significant levels of signaling bias for CCR2, CCR5, and CCR7 in comparisons of G protein activation using several Gα subtypes as well as β-arrestin 2, cAMP, and Ca^2+^ signaling. These recent studies suggest that biased signaling responses to chemokine ligands may be a rather general phenomenon contributing to the different downstream cellular outcomes of chemokine receptor activation.

## 8. Natural and Pharmacological Inhibitors

### 8.1. Viral Chemokines and Receptors

Large DNA viruses such as herpesviruses and poxviruses employ numerous strategies to evade the host immune response. One such strategy is molecular mimicry of chemokines and chemokine receptors to modulate the chemokine signaling network, as described in previous reviews [210,211,212].

Viral chemokines can function as both agonists and antagonists of human chemokine receptors and thereby can promote or inhibit the recruitment of various leukocyte types to infected cells. For instance, viral macrophage inflammatory protein II (vMIP-II or vCCL2), a CC chemokine encoded by Kaposi’s sarcoma-associated herpesvirus (KSHV), is unique in that it is a broad spectrum high affinity ligand of many chemokine receptors from all four subfamilies. vMIP-II is an antagonist of CCR1, CCR2, CCR5, CCR10, CXCR4, CX3CR1, and XCR1 but is an agonist of CCR3 and CCR8, which enables this protein to upregulate the Th2-associated immune response, which is associated with delayed viral clearance [213]. The 3D structure of vMIP-II in complex with human CXCR4 has been determined [14].

Viruses can express receptors that interact directly with human chemokines or that regulate the functions of endogenous chemokine receptors. Several viral chemokine receptors are constitutively active, signaling independently of chemokine ligands and coupling promiscuously to several G proteins. For example, ORF74 is a human CXCR2 homologue encoded by KSHV that is constitutively active, but whose activity can be modulated by endogenous chemokines. Human CXCL1, CXCL2/GROβ/MGSA-β, and CXCL3/GROγ/MGSA-γ act as agonists of ORF74, while human CXCL6/GCP-2, CXCL10, CXCL12, CCL1, and CCL15/HCC-2 as well as vMIP-II act as inverse agonists to control the proliferative signaling potential of ORF74 in virus-infected cells [214,215]. By contrast, BILF1 is a constitutively active orphan receptor that can form heterodimeric complexes with human chemokine receptors and thereby impair chemokine receptor signaling at the G protein level, by scavenging a shared pool of G proteins [216]. Finally, US28 is a CX3CR1 homologue that binds chemokines from both the CC and CX3C sub-families. The crystal structure of US28 bound to a human chemokine (CX3CL1) has been determined [13].

### 8.2. Chemokine-Binding Proteins from Pathogens and Parasites

Large DNA viruses, the parasitic worm *Schistosoma mansoni* [217], and the tick species *Rhipicephalus sanguineus* [218,219] all encode soluble chemokine-binding proteins that disrupt the chemokine signaling network and subsequent activation and recruitment of leukocytes. They do so by hindering the interaction of chemokines with their cognate chemokine receptors and/or GAGs. Most chemokine-binding proteins do not share structural or sequence similarity with chemokine receptors, yet they are sometimes described as soluble chemokine decoy receptors. Many chemokine-binding proteins promiscuously bind many chemokines from multiple sub-families; however, several show specificity for one sub-family. Several detailed reviews of chemokine-binding proteins have been published [220,221,222].

Viral chemokine-binding proteins vary in selectivity for chemokine ligands, but the majority are able to bind chemokines from more than one sub-family. The poxvirus encoded 35-kDa protein is relatively selective in that it binds with high affinity to nearly all CC chemokines and with only low affinity to CXCL8 and CXCL1 [223]. The structure of this 35-kDa protein showed a globular protein with a β-sandwich domain [224,225]. This fold was found to be conserved in several other viral chemokine-binding proteins, including A41 [226], the SECRET (smallpox virus-encoded chemokine receptor) domain of CrmD [227], and the herpesvirus encoded M3 protein [228]. However, A41 and the CrmD SECRET domain interact with a reduced set of both CC and CXC chemokines, while the M3 protein binds promiscuously to chemokines from all sub-families, despite its structural similarity to the 35 k-Da protein. In contrast, the UL21.5 glycoprotein, encoded by human cytomegalovirus has been reported to bind specifically to CCL5, although only a limited number of chemokines were tested [229].

Parasites such as worms and ticks are also known to produce chemokine-binding proteins, presumably allowing them to evade detection and parasitize their mammalian hosts for longer periods. The 36 kDa chemokine-binding protein of *S. mansoni*, smCKBP, is expressed and secreted by *S. mansoni* eggs and binds to a selection of CC and CXC chemokines [217]; the structure of this protein is yet to be determined. The tick species *R. sangunieus* produces three chemokine-binding proteins, named evasins [218,219]. Evasins-1 and -4 bind exclusively to CC chemokines while evasin-3 binds only CXC chemokines. Evasins-1 and -4 differ in selectivity, with evasin-1 binding to three CC chemokines with high affinity and evasin-4 binding to approximately 20 CC chemokines. The structure of evasin-1 has been determined, revealing a novel fold different from that of viral chemokine-binding proteins [230].

### 8.3. Pharmacological Approaches towards Inhibition of Chemokines and Receptors

Considering the importance of the chemokine signaling network in numerous inflammatory and autoimmune diseases, considerable effort has been expended developing both small molecules and biologics (antibodies) that modulate the activity of chemokine receptors or chemokines themselves. Reviews of chemokine receptor antagonists have been published previously [231,232]. Here, we provide a brief overview of the main approaches.

A large number of small molecule chemokine receptor antagonists have been developed. Typically these bind to residues in the transmembrane helices of the receptors, stabilizing the inactive conformation of the receptor and/or preventing binding and activation by chemokines. Two such antagonists have reached the market. The CCR5 antagonist Maraviroc (developed by Pfizer) is used as an antiviral agent in HIV infection to block viral entry into macrophages [233], whereas the CXCR4 inhibitor Plerixafor (AMD3100; developed by AnorMED and marketed by Genzyme) blocks homing of hematopoietic stem cells to the bone marrow, thereby mobilizing these cells to the bloodstream, allowing them to be collected for later transplantation [60].

Small molecule agonists of chemokine receptors have also been discovered. In spite of their size, these agonists are generally able to fully activate chemokine receptors, though not always in the same way as chemokine ligands. For example, the functionally selective CCR5 agonist YM-370749 is able to induce internalization of CCR5 from the cell surface but is unable to induce chemotaxis [234]. Thus, it appears that this compound stabilizes a distinct active conformation of the receptor from that stabilized by chemokine ligands.

While less common, small molecules that bind and antagonize chemokines have also been discovered. For instance, the structure of chemokine CXCL12 in complex with an antagonist was determined, showing that this antagonist occupies the site normally bound by the N-terminal regions of the receptor CXCR4 [235].

Whereas small molecules often have the advantage of high oral bioavailability, they may also suffer from fast clearance rates or relatively low specificity, leading to off-target effects. Therefore, a number of biologics (large biomolecule therapeutics) have also been developed. An anti-CXCL8 antibody is marketed in China for the treatment of psoriasis and antibodies targeting CCL2, CCL5, and CXCL10 are in clinical trials [236,237]. The antibody Mogamulizumab (KW-0761), directed against CCR4 [238], is marketed in Japan for adult T-cell leukemia-lymphoma. Recently, Griffiths et al. have described the interesting example of “i-bodies,” which are human single domain antibodies (human equivalents of shark V_NAR_), that antagonize CXCR4 [239]. Finally, an l-stereoisomer oligonucleotide aptamer targeting CCL2 has progressed to a Phase IIa clinical trials in diabetic nephropathy patients [240].

## 9. Summary and Future Directions

It is widely appreciated that the chemokine-receptor system plays critical roles in immune homeostasis, inflammatory responses, cancer, and several important infectious diseases. The system is inherently complex, consisting of close to fifty chemokines, most of which can activate several receptors, each expressed on a variety of leukocytes and some other cell types. To avoid undesirable inflammation and to ensure appropriate responses to pathogens, this system must be tightly controlled. In this review, we have summarized the major mechanisms that are currently understood to regulate the interactions of chemokines with their receptors.

Although the mechanisms discussed herein together exert substantial biochemical control over the chemokine-receptor network, they also greatly enhance the complexity of the network. For example, post-translational modifications and oligomerization effectively increase the number of chemokines and receptors and vastly expand the number of chemokine-receptor combinations. This presents significant challenges when attempting to inhibit specific targets because inhibitors may not be equally effective against all modified forms of a receptor or chemokine. On the other hand, understanding the mechanisms of regulation also leads to novel opportunities for therapeutic intervention. Thus, in addition to targeting chemokines or receptors directly, it may become possible to influence their activity by indirectly suppressing their expression, altering their localization or blocking downstream signaling pathways. Ongoing studies, such as those reported in this special issue of the *International Journal of Molecular Sciences*, are continuing to reveal novel aspects of chemokine-receptor regulation and to stimulate new approaches to pharmacological control.

## Abbreviations

AP2	Adaptin 2
CKBP	Chemokine-binding protein
DC-CK	Dendritic cell-derived CC chemokine
ENA	Epithelial-derived neutrophil-activating
GCP	Granulocyte chemotactic protein
GRO	Growth-regulated protein
IL	Interleukin
IP	Interferon γ-induced protein
IUPHAR	International Union of Basic and Clinical Pharmacology
I-TAC	Interferon-inducible T-cell α chemoattractant
MCP	Monocyte chemoattractant protein
MGSA	Melanoma growth stimulating activity
MIP	Macrophage inflammatory protein
PARC	Pulmonary and activation-regulated chemokine
PF4	Platelet factor 4
RANTES	Regulated on activation, normal T cell expressed and secreted
SDF	Stromal cell-derived factor
SECRET	Smallpox virus-encoded chemokine receptor
SLC	Secondary lymphoid tissue chemokine
SNP	Single nucleotide polymorphism
TARC	Thymus and activation regulated chemokine
TECK	Thymus-expressed chemokine
